# Cell Cycle Modulation of CHO-K1 Cells Under Genistein Treatment Correlates with Cells Senescence, Apoptosis and ROS Level but in a Dose-Dependent Manner

**DOI:** 10.15171/apb.2019.054

**Published:** 2019-08-01

**Authors:** Riris Istighfari Jenie, Nur Dina Amalina, Gagas Pradani Nur Ilmawati, Rohmad Yudi Utomo, Muthi Ikawati, Annisa Khumaira, Jun- Ya Kato, Edy Meiyanto

**Affiliations:** ^1^Department of Pharmaceutical Chemistry, Faculty of Pharmacy, Universitas Gadjah Mada, Yogyakarta 55281, Indonesia.; ^2^Cancer Chemoprevention Research Center, Faculty of Pharmacy, Universitas Gadjah Mada, Yogyakarta 55281, Indonesia.; ^3^Laboratory of Tumor Cell Biology, Graduate School of Biological Sciences, Nara Institute of Science and Technology, Ikoma, Nara, 630-0101, Japan.

**Keywords:** Genistein, Cell cycle, Senescence, Apoptosis, Reactive oxygen species (ROS)

## Abstract

***Purpose:*** Genistein, a soy isoflavone, exhibits a biphasic effect on cells proliferation with some different effects between ER-alpha and ER-beta. The objective of this present study is to determine the modulatory effect based on cell cycle progression under genistein treatment in combination with 17-β estradiol (E2) on CHO-K1 cells.

***Methods:*** The effect of genistein 0.1-100 µM on cells proliferation was examined by MTT assay. The modulation of genistein and estradiol (E2) on cell cycle and apoptosis were observed by using flowcytometry with PI and PI/AnnexinV staining, respectively. Moreover, the effect of genistein and E2 on senescence cells, and ROS level were determined by senescence-associated β-galactosidase (SA β-gal) staining and by using flowcytometry with 2’, 7’–dichlorofluorescin diacetate (DCFDA) staining, respectively. The expression level of the cell cycle and senescence protein markers were observed by immunoblotting.

***Results:*** Single treatment of genistein at physiologically achievable (low) concentration (<2 µM) induced proliferation of CHO-K1 cells while at a pharmacological (high) concentration (50 and 100 µM) suppressed cells proliferation. Interestingly, treatment of genistein at the physiological concentration in combination with E2 for 24, 48 and 72 h decreased cells viability on CHO-K1 cells compared to untreated cells. Further analysis of the cells showed that 50 µM genistein induced G2/M phase accumulation and induced apoptosis. Moreover, genistein induced cell senescence and increased ROS level. Immunoblotting analysis showed the decreasing of ERalpha, Bcl2, and ppRb protein level upon treatment of 1 µM Gen and 1 nM E2.

***Conclusion:*** Our results suggest that the cell proliferation inhibitory mechanism of genistein at pharmacological concentration involved the induction of cell senescence, and the elevation of ROS level. Moreover, the decreased of cells proliferation upon treatment of physiological concentration of genistein in combination with E2 may be correlated with the alteration of ER expression.

## Introduction

The proliferation of breast cancers and ovarian cancers usually involves estrogen receptor (ER) pathway in addition to being driven through mitogen-activated protein kinases (MAPKs) signal transduction.^1,2^ This pathway will be active when estrogen binds to and activate its receptor, which further activates the expression of genes necessary to spur cell cycle progression. This pathway commonly occurs in ER positive breast cancers.^3,4^ Therefore, the development of anti-cancer agents with the biphasic phenomena, such as genistein, needs to consider the specificity of the ligand-receptor interaction to gain the effectiveness of the treatment. On the other hand, ERs are also needed in several important physiological processes in the body, such as the growth of primary and secondary female sexual tissues, bone integrity, and cholesterol metabolism.^5^ Therefore, ER pathways are also very essential, especially to maintain women’s health. Disturbance in this pathway may cause the inherent physiological imbalance in body.^6^ To address this concern, the development of an anti-cancer agent targeted at the protein kinases that may also affect the function of ERs should be given special attention by providing more detailed information of its selectivity following its use later.

Genistein is a natural origin compound that found in large quantities of soybean crops. Genistein is capable of inhibiting the growth of cancer cells through various mechanisms.^7^ Genistein inhibits cell cycle and modulates the expression of some proteins involved in the cell cycle at the concentrations of 5-200 μM.^8^ Besides inhibiting the cell cycle, genistein was known to induce apoptosis in various epithelial cancer cells. The apoptotic effect of genistein appears in the concentration range of 10-200 μM. Genistein treatment may also affect the activation of protein kinase B (Akt) and nuclear factor kappa B (NF**κ**B). In inhibiting cancer metastases, genistein inhibits cell migration and decreases the expression of matrix metalloproteinases (MMPs) that plays a role in the degradation of extracellular matrix.^7^ All of the data exert genistein as a potential anticancer agent.

In addition to the capability of inhibiting the growth of cancer cells, genistein is also able to show estrogenic effects to induce proliferation in ER-expressing cells. Moreover, genistein at low concentrations, i.e. 0.1-10 μM inhibits bone matrix degradation process and increases the bone formation.^9^ Therefore, Genistein on certain levels can be applied for anti-osteoporosis due to its estrogenic effect. Genistein performs good binding to both ER-alpha and ER-beta, but the interaction to each receptor exhibit difference effect. Genistein likely to be estrogenic through ER-alpha, but it becomes anti-estrogenic through ER-beta.^10^ Therefore, when using genistein, we should consider where the tumor cells reside because the breast and the ovary exhibiting difference type of ER expression.

The ovarium cells dominantly express ER-beta rather than ER-alpha^11^ that may lead to the different response of genistein. Moreover, the binding affinity of genistein to ER-beta is 20 fold higher than to ER-alpha.^12^ In this concern, we explore the effect of genistein on the CHO-K1 focusing on cell proliferation, apoptosis, cells senescence, and its molecular mechanism. CHO-K1 is an immortalised cell line derived from ovarian tissue, which is characterised by high expression of ER-beta over ER-alpha.^13^ Our report showed biphasically, the estrogenic and anti-estrogenic, effect of genistein and its physiological changes at a dose-dependent manner that may be important to be considered when using genistein especially at the high concentration.

## Materials and Methods

### Cell culture

Chinese hamster ovary (CHO-K1) cell line used in this study were kindly provided by Professor Masashi Kawaichi (Nara Institute of Science and Technology, Japan). Briefly, CHO-K1 cells were cultured in Roswell Park Memorial Institute (RPMI) 1640 (Gibco, USA) supplemented with 10% (v/v) fetal bovine serum (FBS) (Sigma, USA), HEPES, sodium bicarbonate, 150 IU/mL Penicillin, 150 μg/mL Streptomycin (Gibco) and 12.5 μg/mL Amphotericin B (Gibco). Cells were grown at 37°C with 5% CO2 in a humidified atmosphere.

### MTT cytotoxicity and proliferation assay

The cytotoxicity of genistein and E2 was tested using 3-(4,5-dimethyl thiazol-2-il)-2,5-diphenyltetrazolium bromide (MTT) assays. CHO-K1 cells (3 × 10^3^) were seeded in 96-well microplate and cultured for 24 hours. Cells were treated with genistein (0.1-100 μM) and in combination with E2 (0.1 and 1 nM) at 24, 48, and 72 h. Untreated cells were regarded as negative controls. After treatment, cells were treated with 0.5 mg/mL of MTT (Biovision) and incubated further for four hours. Cells were lysed using sodium dodecyl sulfate (SDS) stopper containing 0.01 N HCl and incubated in the dark condition overnight. After incubation, the absorbance was measured by ELISA reader plate at λ 595 nm. The absorbance was converted to % cell viability by comparing the treated group with the untreated at particular time course. Linear regression between concentration and % cell viability giving the equation y = Bx + A were used to calculate IC_50_ value, that was the concentration inhibiting 50% cell proliferation. In the experiments to measure proliferation rates, 3 × 10^3^ CHO-K1 cells were seeded in 96-well microplate as described above and treated with genistein and its combination with E2 at several concentrations for 24, 48, and 72 hours before MTT assay.

### Cell cycle assay

The cell cycle distribution after treatment was evaluated by flow cytometry. Cells (2.5 × 10^5^) were grown in 6-wellplate 24 h prior to the treatment. The cells were treated with genistein, E2, and its combination and incubated for 24 h and 48 h. The next day, all the medium was discarded in conical, the cells were trypsinized and centrifuged 2000 rpm for 3 minutes. Cell pellets collected were then fixed with ethanol for 30 minutes and centrifuged again for 3 minutes. Cells then were washed twice with cold PBS and centrifuged again before resuspended in propidium iodide solution (50 μg/mL in PBS containing 1% triton X-100) and treated with DNase-free RNase A (20 μg/mL) for 30 minutes at 37°C in dark place. Treated cells then were injected to BD C6 Accuri Flowcytometry (BD Bioscience). The red fluorescence was measured using the FL-1 setting (log mode) after the cell debris was electronically gated out. Two hundred thousand events were acquired and analysed by Modfit LT^TM^ (Verity Software House).

### Apoptosis assay

The apoptosis induction of genistein and E2 was determined by flow cytometry. Cells (2.5 × 10^5^) were grown for 24 h then treated with genistein, E2 and the combination for 24 h and 48 h. After the treatment, the medium was discarded, and cells were harvested by using trypsinization. Cells were stained with Annexin-V-FLUOS staining kit (Roche) consisting of 100 mL of binding buffer, 2 mL Annexin-V and 2 mL of propidium iodide (PI), and incubated for 10 minutes in the darkroom, according to manufacturer’s instruction. Cells were then analysed by BD C6 Accuri Flowcytometry (BD Bioscience).

### Senescence-associated β-galactosidase assay

Senescence-associated β-galactosidase (SA β-gal) staining was performed as previously described. CHO-K1 cells were seeded at 1×10^4^ cells in 6-well plate. The SA-β-gal staining was performed 24 after seeding when cells were normally in log phase growth and 60%–70% confluent, to minimize false-positive staining. For SA-β-gal staining, cells were washed twice with PBS 1x and fixed for 10 minutes with 2% formaldehyde-0.2% glutaraldehyde. After washing again with PBS 1x, CHO-K1 cells were incubated overnight at 37°C CO_2_ free in staining solution containing 0.2% 5-bromo-4-chloro-3-inolyl-β-D-galactoside, 40 mM citric acid/phosphate buffer (pH 6.0), 5 mM K_4_Fe(CN)_6_, 5 mM K_3_Fe(CN)_6_ and 2 mM MgCl_2_. Cells were observed under inverted microscope (100x magnification).

### ROS assay

A total of 5×10^4^ CHO-K1 cells were grown on 24 well plates with DMEM culture medium (Gibco) overnight. Cells were harvested with trypsin-EDTA 0.25% (Gibco), then added 500 μL 1x supplemented buffer for inactivation of trypsin. Cells were stained using a 25 μM 2’, 7’–dichlorofluorescin diacetate (DCFDA) (Sigma) then were incubated at 37°C CO_2_ 5% for 30 min. Each cell were treated with genistein and E2, or H_2_O_2_ then were incubated at 37°C CO_2_ 5% for 4 h. Intracellular ROS was analyzed BD Accuri C6 flowcytometry (BD Bioscience).

### Immunoblotting assay

Approximately 7×10^5^ CHO-K1 cells were seeded on 10 cm tissue culture dish and incubated for 24 hours. Cells were treated with genistein, E2 in a single treatment and its combination for 24 hours. Protein was extracted using radioimmunoprecipitation assay (RIPA) buffer (Tris HCl pH 7.6, NP 40, Na deoxycholate, NaCl, SDS, phenyl methyl sulfonyl fluoride (PMSF), NaF, and cocktail inhibitor protease), then separated in 10% acrylamide gel (ER, Rb and β-actin) and 14% acrylamide gel (Bcl-2) by SDS-PAGE electrophoresis. After transferred to polyvinylidene fluoride (PVDF) membrane, the membrane was incubated overnight at 4°C with either the mouse monoclonal antibody against ER alpha (ER-alpha) (Santa Cruz sc-542), Retinoblastoma (Rb) (BD Bioscience), β-actin (Santa Cruz sc-47778), B cell lymphoma 2 (Bcl-2) (BD Bioscience-Cat. 519002078). After incubation with secondary antibody anti-mouse (Santa Cruz sc-516102) for 1 hour, the protein bands were visualized using enhanced chemiluminescence (ECL) (BioRad) and detected using Luminograph (Atto). The relative protein levels were calculated as a ratio with the amount of β-actin protein.

### Statistical analysis

Statistical analysis was performed using Student’s t test (Excel 2013 software; Microsoft, Redmond, WA). *P* values less than 0.05 were considered significant. Immunoblotting results were quantified by using ImageJ software (National Institutes of Health, Bethesda, MD).

## Results and Discussion

Genistein is an isoflavone, found robustly in soybean, already studied as a chemopreventive agent through various pathways.^7^ The previous study showed that genistein performed biphasic effect toward ER expressing cancer cells which induce proliferation of MCF7 cells in a low concentration but suppressed cells growth at a high concentration.^14^ Further study revealed that genistein exhibits similar physiological effect with estrogens, such as regulating cholesterol metabolism, bone remodelling, and breast gland epithelial cells development.^15^ However, in some cases, genistein also modulates MAPK signalling pathways leading to modulation of cells proliferation. Thus, in this study, we investigated the genistein effects in CHO-K1 cell proliferation, cell cycle, apoptosis, ROS expression and cell senescence in the combination with estradiol (E2).

### Cytotoxic and proliferation effect of genistein in single and combination on CHO-K1 cells

**Figure 1:**
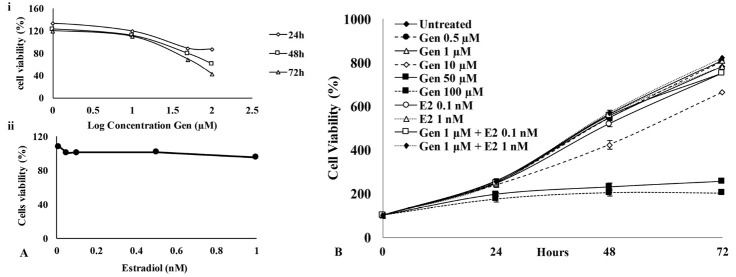
**Genistein showed biphasic effects in single and in combination with estradiol on the proliferation of CHO-K1 cells.** Cells (3 × 10^3^) were treated with different concentrations of genistein (A-i) or E2 (A-ii) as indicated in the graph. B. Cells also treated with single genistein (low and high concentration), single E2 (0.1 and 1 nM), or combination of low concentration genistein and E2 for 0, 24, 48, and 72 h (B). Cytotoxic activity and proliferation assay were determined by MTT assay as described in the Materials and Methods. Error bar represents standard deviation (*n* = 3, *P < 0.05 by Student’s *t* test).

Since genistein is well known to possess biphasic effect, especially on ER expressing cells, it is important to explore deeper the specific physiological phenomenon in relation to the doses and the variation time course of treatment. We employed CHO-K1 cells, which are known to express both ER alpha and beta mRNA.^13^ First, we evaluated the effect of genistein in various concentrations and incubation time on the cells viability by performing the MTT assay. The results showed that genistein in single treatment exhibit biphasic effect on the cells, showing that at low concentration (<1 μM), genistein stimulated cells growth up to >200 %, while at high concentration (>10 μM), genistein significantly decreased the cells viability with the degree of depletion in a time-dependent manner (Figure 1A-i). In this regard, genistein showed a strong inhibitory effect at the concentration of 50 μM.

Although the low concentration of genistein generally induces proliferation on CHO-K1 cells, there is a study reported that low concentration of genistein in combination with estrogen (E2) inhibits MCF-7 cells proliferation.^15^ Based on that report, we examined the effect of low concentration of genistein in combination with E2 in CHO-K1 cells. As a confirmation, we examined the effect of 0.1 and 1 nM E2 on the tested cells that brought no effect in CHO-K1 cells proliferation after 24 h of treatment (Figure 1A-ii). Interestingly, the proliferation profile altered after 48 h. Treatment with a higher concentration of E2 tend to elevate the cell viabilities up to 72 h, but not in lower concentration compared to untreated cells. This fact demonstrated that a different concentration of E2 gave different cell proliferation effects. Overall, the single treatment in a low concentration of genistein and E2 (0.1 and 1 nM) modulate CHO-K1 cells proliferation. Subsequently, E2 was combined with genistein to evaluate whether the combination initiates the cells proliferation or inhibit the cells viability. Interestingly, treatment of genistein at low concentration in combination with E2 reduced cells viability in 48 h and 72 h (Figure 1B). The effect of genistein on CHO-K1 cells might be modulated through the cell cycle and apoptosis modulation.

Our results showed the biphasic effect of genistein towards CHO-K1 cells in a time-dependent manner as shown in Figure 1. We found that genistein in combination with E2 decreased cells viability (Figure 1B). One explanation is that the decrease in cell viability may be due to the presence of unfunctional ER-alpha despite the role of ER-beta in CHO-K1 cell proliferation.^16^ In addition, genistein may act through the other pathways such as that genistein in the occurrence of low concentration of E2 may cause cell proliferation attenuation through MAPK pathways.^17^ Since the MAPK pathways play in different responses to the cells such as proliferation, differentiation, development, inflammation and apoptosis, this effect straightly could affect the cell viability. Another report said that ERK 1/2 activity also modulated by genistein treatment related to its activity on cell proliferation.^18^ However, this phenomenon by which genistein in combination with E2 suppresses cell growth through MAPK inhibition should be clarified further.

### Cell cycle modulation and apoptosis induction in response to genistein and E2 treatment

The MAPK cascades brought various responses to the cells. Recent studies reported that genistein could alter the upstream and downstream expression of MAPK pathways.^19^ This phenomenon resulted in cell cycle modulation and apoptosis profile of the endometrium cancer cell. Thus, here we also examine the cell cycle and apoptosis profiles to understand the genistein and E2 combination of CHO-K1 cells proliferation.

**Figure 2:**
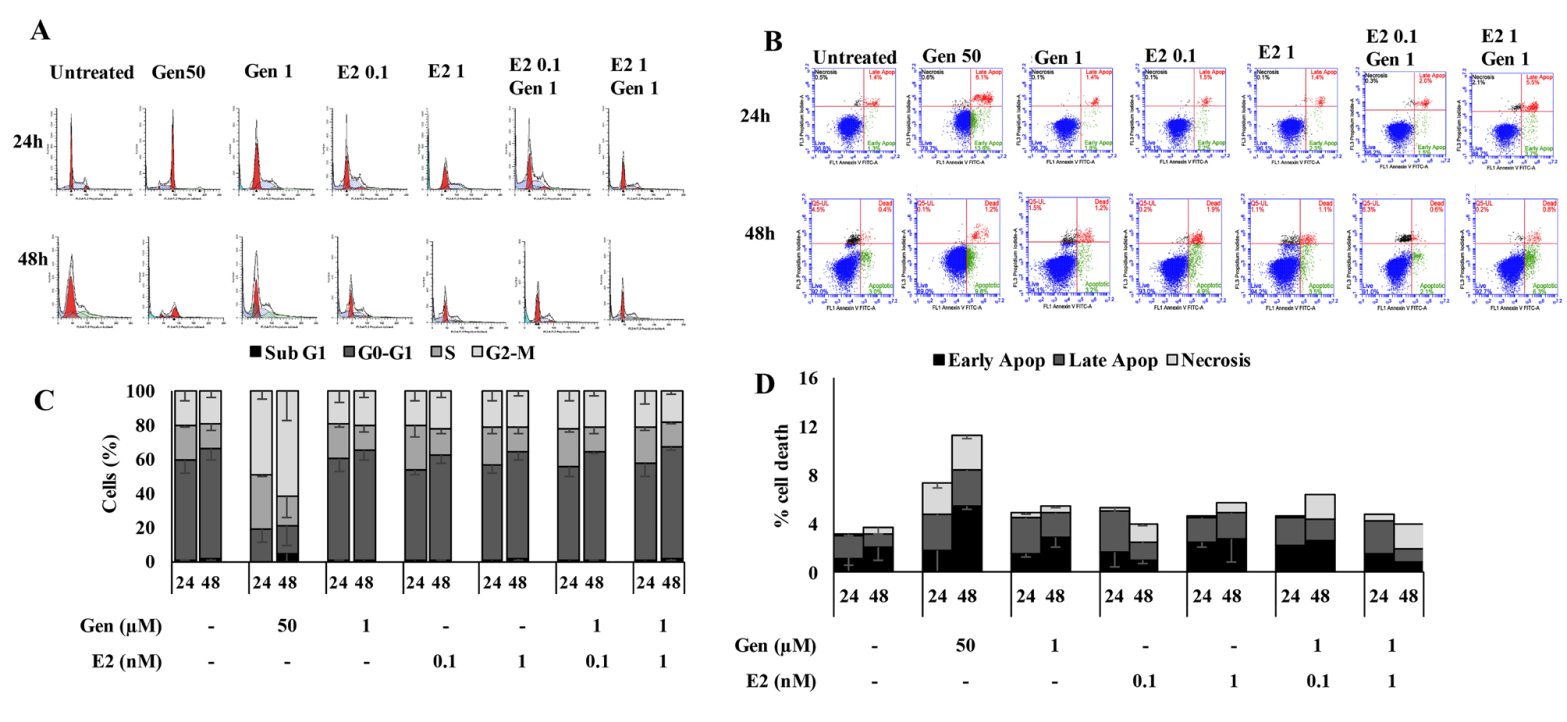
**High concentration of genistein induced G2/M arrest and apoptosis of CHO-K1 cells.** Cells (2.5 × 10^5^) were treated for 24 and 48 hours with genistein, E2, or its combination with concentration as indicated on the graph. Cells were then stained with propidium iodide/RNase for cell cycle analysis (A) or stained with PI/Annexin V for apoptosis analysis (B). Cells were then analyzed using BD C6 Accuri Flowcytometry (BD Bioscience) (C and D) as described in the Materials and Methods.

We explored the effect of genistein in single and in combination with E2 (at low and high concentration) on the physiological changes focusing on cell cycle (Figure 2A and C) and apoptosis profiles (Figure 2B and D). In this study, treatment of CHO-K1 cells with a low concentration of genistein (1 µM) as well as E2 did not change the cell cycle profiles compared to the untreated in both treatments for 24 and 48 hours. Moreover, the combination treatment also showed a similar profile, including the combination with 1 nM E2, seemed did not increase cell accumulation at Sub G0/G1 and G1 phase. In contrast, the treatment of genistein at high concentration (50 µM) induced cell cycle accumulation at G2/M phase and sub G0/G1, indicated that the cells were induced into apoptosis or necrosis via G2/M arrest. According to this result (Figure 2A and C), the changes of cells viability in response to genistein at low concentration and in combination with E2 seems irrespective to the cell cycle progression.

This programmed cell death mechanism brought to eliminate the damaged cells and reduce the cells population. Our result (Figure 2B and D) showed that the percentages of apoptotic cells under treatment of low concentration genistein in single or in combination with E2 (at low and high concentration) for 24 and 48 hours did not induce apoptosis. In contrast, treatment with high concentration of genistein (50 µM) significantly induced apoptosis. (Figure 2B). In conclusion, the modulation of cells proliferation depends on the specific treatment in correlation with apoptotic evidence. We suggest that alteration in particular treatment of cell proliferation happened through another biological process.

The phenomenon was also shown in our result of the cell cycle and apoptosis assay (Figure 2). We found that the single treatment of genistein at high concentration increased cells number in the G2/M phase and Sub G0/G1 (Figure 2A and C). Similar results were performed by previous studies that had been reported^20-22^ which showed genistein could inhibit the growth of HT-29, Caco-2, and HCT-116 cells by an accumulation of cells at the G2/M phase. However, the low concentration of genistein and E2 did not induce cell cycle arrest. The combination treatment also showed the same phenomenon but the combination with 1 nM E2 seemed to increase Sub G0/G1 and G1 phase. These results are in line with previous studies, which reported that genistein in low concentration (20 nM) did not induce cell cycle arrest in HCT-116 cells.^22^ Even the combination treatment causes decreasing cells viability without changing on the cell cycle profiles, this contrast phenomenon brought us into a challenging case to be elucidated. These findings showed that genistein at high concentration could potentially inhibit cell proliferation. Thus, it is prospective to be developed as an anticancer agent. Moreover, we have to pay attention to consuming genistein-containing products.

This finding was interesting as there was a high possibility that genistein might cause induction of apoptosis as there was an increasing number of cells arrest in sub G0/G1, especially at the high concentration. Thus, the apoptosis assay was also carried out in this present study to evaluate whether or not the inhibitory effect by genistein in the presence of E2 was the reason of its activity towards ER or induction toward apoptosis. Based on the experiment (Figure 2B and D), the treatment with 50 µM genistein for 24 and 48 h alone increased apoptotic cells compared to the untreated, meanwhile single E2, low concentration of genistein, and its combination treatment did not give any increasing of apoptotic cells for 24 h even also for 48 h. This finding is in contrast with other group findings who reported that estrogen and estrogen-like compounds (E2) induced anti-proliferation and apoptosis effects in Hep3B cells and that the E2 and E2-like compounds mediated apoptotic effect is ER-dependent.^23^ It has been reported that 1 µM genistein plus 1 nM E2 significantly increased apoptosis with a concomitant decrease in ERK1/2 phosphorylation.^24^ High concentration of genistein (100 µM) both in the presence and absence of E2 also increases apoptosis of MDA-MB-231 breast cancer cells.^18^ This unique phenomenon might be due to the competitive evidence between E2 and genistein to ER leading to abrogation of ER activation.^15^ However, we should consider that physiological alteration in the cells under the disruption of intracellular signalling by this treatment could also be through the other specific mechanism based on the antioxidant or oxidant properties of genistein.

### Senescence induction by genistein and its combination with E2 on CHO-K1 cells

**Figure 3:**
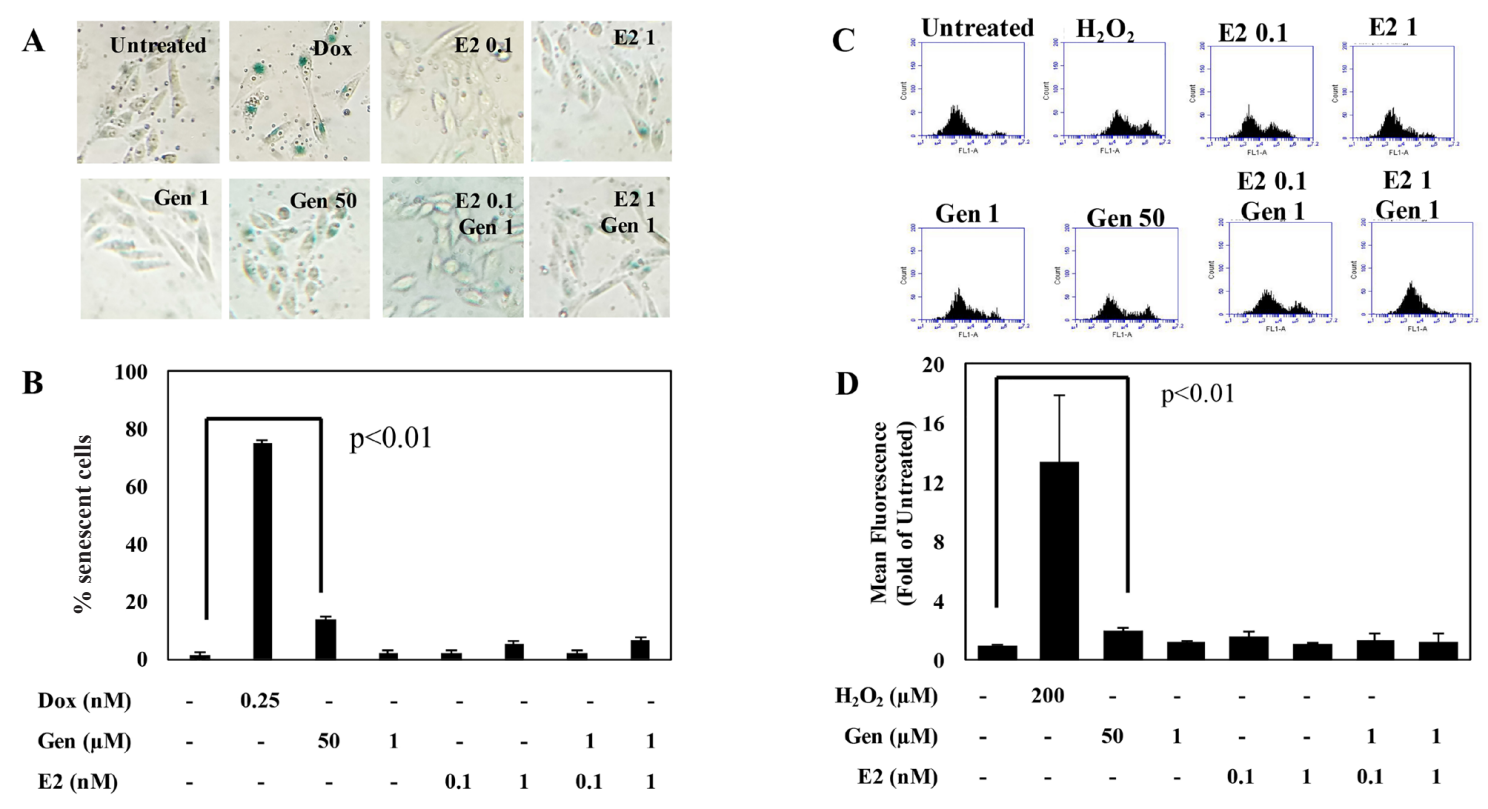
**High concentration of genistein induced cell senescence and increased ROS level.** Cells (1×10^4^ ) were grown as described in the Materials and Method section, then treated for 24 h with doxorubicin (Dox), genistein (Gen), estradiol (E2), and the combination of Gen-E2 with concentration as depicted in the graph. Cells were then stained by X-gal solution for 48 h resulting in blue-green colour cells. Senescence evidence was monitored by an inverted microscope in 100x magnification (A) and quantified (B). Cells (5×10^4^) were grown for 24 h then harvested. Cells were then stained by DCFDA fluorescence dye for 30 minutes and were treated with hydrogen peroxide (H_2_O_2_), Gen, E2, and the combination of Gen and E2 for 4 h, then were subjected for ROS detection by using BD C6 Accuri Flowcytometry (BD Bioscience) (C). Total ROS level after a single treatment of genistein and combination with E2 on CHO-K1 cells (D).

Senescence is physiological proses that affect the cells viability in addition to cell cycle arrest and apoptosis which is induced by ROS level and increasing level of several anti-apoptotic proteins.^25^ Subsequently, the senescence evidence under a combination treatment of genistein and E2 needs to be confirmed. Therefore, we used SAβ-gal staining assay to examine senescence evidence, which represented as blue-green colour cells. The results showed that single treatment of 1 µM genistein did not induce senescence, in contrast with 50 µM genistein, which induced significantly (Figure 3 A and B). Combination of genistein 1 µM with E2 0.1 and 1 nM slightly induced senescence after 24 h treatment, however, this effect similar with the treatment of E2 alone suggesting that the effect in the combination may be due to E2 action (Figure 3 A and B). These results confirmed that the decreasing of cells viability correlated with senescence induction.

One of the important factors regulating senescence is the cellular reactive oxygen species (ROS) production. In this concern, we performed oxidized DCFDA staining flow cytometry to measure ROS level intracellular on CHO-K1 cells. Our result showed that single treatment of low concentration of genistein (1 µM), E2 (0.1 and 1 nM), and the combination of both slightly increased, but not significantly, the ROS level compared to the untreated (Figure 3 C and D). In accordance with the data of senescence assay, treatment of 50 µM genistein increased ROS level compared to untreated cells.

Cell cycle arrest is only a part of the equation of senescence.^26^ Recently, senescence is considered to be an integrated and a widespread component that holds an important aspect in tumor development, tumor suppression and the response to therapy.^27^ Therefore, in this study, it becomes interesting to observe senescence especially in the irregular phenomenon of genistein treatment at low concentration in combination with E2 0.1 and 1 nM that reduced cells viability but only slightly induced cell cycle arrest and apoptosis after 24 and 48 h. The different phenomenon occurs in the high concentration of genistein, which increased ROS level and induced cells senescence. Therefore, the decline in cell viability after 48 h of treatment with high concentration of genistein was caused due to cell senescence and correlated with the increasing ROS levels. However, this phenomenon should be investigated further.

Numerous studies suggested that ROS may also participate in senescence.^28-30^ The increase of ROS level have been demonstrated as potentially critical for the induction of cell senescence process.^31^ High ROS production is one of the characteristics of proliferative cells that has been considered to be a target of anti-oxidant substances. ROS was considered to induce cellular senescence.^32^ Our data showed that genistein at high concentration (50 μM) elevated ROS level (Figure 3C and D) and this mechanism leads to cells senescence (Figure 3A and B). The previous study showed that genistein induced ROS level expression through SOD down-regulation in HT-29 cells.^20^ Moreover, we observed the higher ROS induction caused by genistein treatment in NIH 3T3 cells than in CHO-K1 cells (data not shown). Similar to this result, ROS expression modulated by genistein also up-regulated in different cells ,i.e. irradiated A549 cells (lung cancer cells) and MRC-5 cells (lung fibroblast cells).^33^ It has been well understood that each cell from different tissue possessed diverse protein active content that mediates the different function of the tissue. The finding that genistein promoted ROS might be similar to that of E2 due to its mitogenic function that is ER-dependent.^32^ It has been reported that in the CHO-K1 cells, ROS expression is mediated through SOD down-regulation.^34^ Our findings suggesting that one of the cell viability suppression was initiated by inducing oxidative stress.

### ER-alpha, Bcl-2, and Rb expression of CHO-K1 cell under genistein and E2 treatment

**Figure 4:**
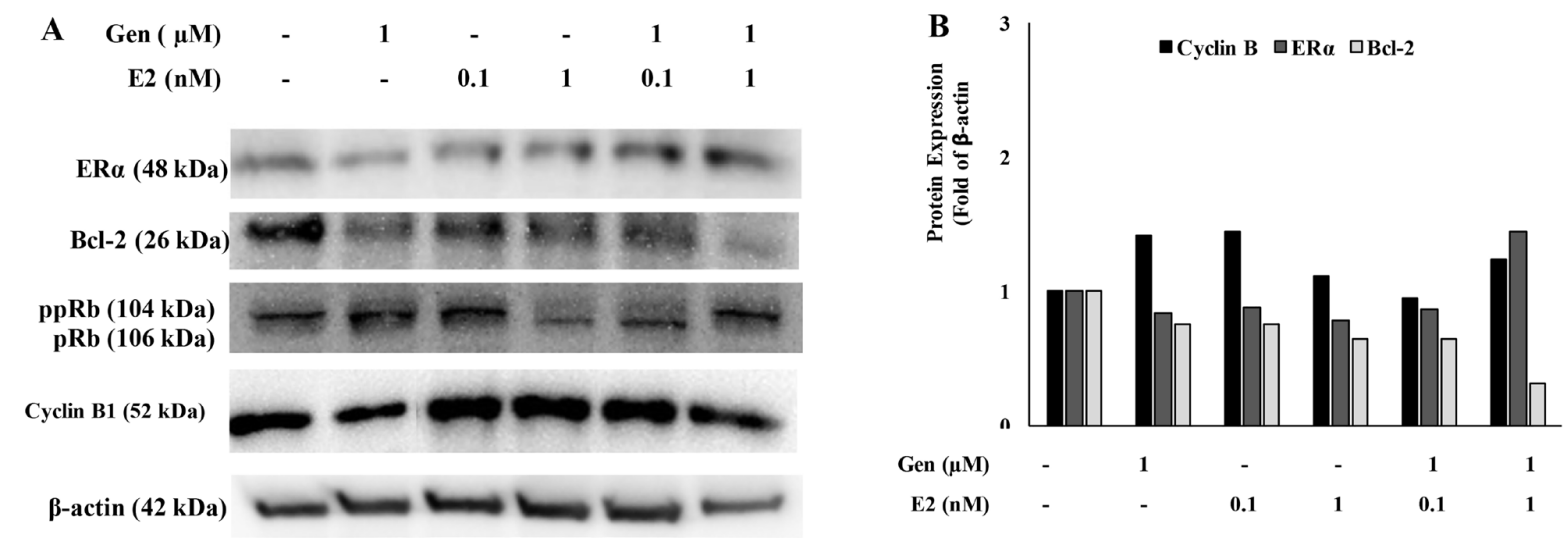
**Protein expression of apoptosis and senescence marker under treatment of genistein and E2 in CHO-K1 cells.** Cells (7×10^5^) were grown and treated with 1 µM genistein (Gen), 0.1 and 1 nM estradiol (E2), and combination of both for 24 h then subjected to immunoblotting assay with antibodies as shown in the picture (A). he protein bands were then quantified by using ImageJ software (B).

To confirm the result of apoptosis and senescence assay, we conducted protein immunoblotting assay to know the marker proteins expression in apoptosis and senescence to understand the molecular level modulation according to the treatments. A single treatment of genistein and E2 practically down-regulated almost all of the protein expression. Genistein in combination with the low and high concentration of E2 showed different protein expression pattern (Figure 4). While the combination of genistein and 0.1 nM E2 treatment down-regulated most of all protein expression compared to the untreated. However, a high concentration of E2 (1 nM) in combination with 1 μM genistein, suppressed the Bcl-2 and up-regulated the ER-alpha expression compared to single treatment and untreated cells. This result showed that the combination of genistein and E2 lead to the different molecular response due to E2 concentration in the cell. These mechanisms appeared to be the key reason for the decline in cell viability under single and combination treatments (Figure 1).

We also confirmed the cells senescence by investigating the changes in some protein expressions such as pRb, ppRb, and Bcl-2. Interestingly, our results showed that genistein treatment at low concentration and the combination with E2 down-regulated Bcl-2 and ppRb. Otherwise, it has been well understood that genistein treatment at a high concentration overexpressed the pro-apoptosis protein.^35-38^ Moreover, the high level of hypo-phosphorylated pRB protein acts as a negative regulator of the mitotic cell cycle resulting in cell cycle arrest. The status of phosphorylated pRb (ppRb) shall be up-regulated by ER expression with cyclin D involvement in the pathway to induce cell cycle progression. In this context, we found a different phenomenon of E2 at high concentration and at low concentration involved in the effects of low concentration of genistein treatment to the pRb status. In this regard, a higher concentration of E2 probably enhanced apoptosis induction.

We assumed that genistein plus E2 treatment have a different mode of action in respect to its concentration in the cells causing cells senescence, even more leading to cell apoptosis at a particular concentration and the status of phosphorylated pRb plays an important role. Moreover, we found that Cyc B level was relatively not changed under treatments (Figure 4), indicated that low concentration of genistein did not cause G2/M arrest. This data were inline with the previous data from cell cycle analysis (Figure 2 A and 2C). However, treatment with the high concentration of genistein showed cell cycle arrest at G2/M phase (Figure 2 A and 2C) and this probably correlated with the modulation of Cyc B expression.^21^ Based on these results, genistein, at the high concentration, induced cell cycle arrest in correlation with the apoptosis evidence and may be other physiological processes such as autophagy. The underlying mechanism needs to be explored based on particular time course.

As the sum up of this study, we testify that genistein exhibit a biphasic effect on CHO-K1 cells. At a high concentration (50 µM), genistein caused cell cycle arrest at G2/M phase and induced apoptosis, but at the low concentration (<2 µM) genistein enhanced cells proliferation. The combination of genistein and E2 in low and high concentration did not modulate cell cycle progression but decrease cell viability. In-depth, this combination induced cells senescence but not apoptosis. At the high concentration of genistein decreased cell viability through induction of cell cycle arrest, apoptosis and ROS level but not senescence. One of the causes of cell senescence is due to the role of ROS level. Overall, the data are impressive that the high hormone condition, the consumption of genistein, such as consumption of soy product increases the risk of cellular disruption, especially for ER expressing cells that may lead to the ageing process. On the other case, lower doses of genistein from soy foods would be expected to act similarly to estrogens with a beneficial effect on ER-expressing tissues, but at high doses, genistein has potentially as anticancer property. All of the findings in this study bring us to a new insight on developing genistein or genistein containing products for some particular applications.

## Conclusion

This study demonstrated that genistein exerts a biphasic effect on CHO-K1 cells. Genistein at the low concentration in combination with E2 at low concentration inhibited ovarian cell proliferation through senescence, apoptosis induction and ER protein expression modulation as well as phosphorylated pRb status. Genistein at high concentration inhibited ovarian cell proliferation through G2/M cell cycle modulation, apoptosis induction, ROS elevation and senescence induction.

## Ethical Issues

Not applicable

## Conflict of Interest

We declare that we have no conflict of interest.

## Acknowledgments

We thank The Ministry of Research and Technology, Higher Education, Republic of Indonesia who provides the grant under the competence-based grant program 2016-2018.

We thank Prof. Masashi Kawaichi from Nara Institute of Science and Technology (NAIST) for his kind gift of CHO-K1 cells.
